# Alk1 haploinsufficiency causes glomerular dysfunction and microalbuminuria in diabetic mice

**DOI:** 10.1038/s41598-020-68515-z

**Published:** 2020-08-04

**Authors:** Cindy Lora Gil, Nathalie Henley, François A. Leblond, Naoufal Akla, Louis-Philippe Laurin, Virginie Royal, Casimiro Gerarduzzi, Vincent Pichette, Bruno Larrivée

**Affiliations:** 1 Department of Biomedical Sciences, University of Montreal, C.P. 6128, Succursale Centre-Ville, Montréal, QC H3C 3J7 Canada; 2 Maisonneuve-Rosemont Hospital Research Centre, University of Montreal, 5415 boul. L’Assomption, Montréal, QC H1T 2M4 Canada; 3 Department of Ophtalmology, University of Montreal, C.P. 6128, Succursale Centre-Ville, Montréal, QC H3C 3J7 Canada

**Keywords:** Mechanisms of disease, Diabetic nephropathy

## Abstract

Endothelial dysfunction has been shown to play an important role in the pathogenesis of glomerular damage during diabetic kidney disease (DKD). As such, a better understanding of the molecular mechanisms involved in glomerular endothelial dysfunctions could provide novel therapeutic strategies for the prevention of DKD. We have previously shown that Alk1/BMP9 signaling plays an important function to maintain vascular integrity in diabetic animals. As such, we evaluated the effects of Alk1 suppression on glomerular endothelial function in diabetic mice. In the present study, we used mice with conditional heterozygote deletion of Alk1 in the endothelium (Alk1ΔEC) to evaluate the role of Alk1 on kidney function during STZ-induced diabetes. DKD was investigated in diabetic control and Alk1ΔEC mice euthanized eight weeks after the onset of diabetes. We showed that Alk1 expression is reduced in the glomeruli of human DKD patients. While renal function was not altered in Alk1ΔEC non-diabetic mice, we showed that Alk1 haploinsufficiency in the glomerular endothelium leads to microalbuminuria, thickening of the glomerular basement membrane, glomerular apoptosis and podocyte loss in diabetic mice. These data suggest that Alk1 is important for the proper function of glomerular endothelial cells and that decreased Alk1 combined with chronic hyperglycemia can impair renal function.

## Introduction

Diabetes is the leading cause of end-stage kidney failure in the developed world^1^. Diabetic kidney disease (DKD) is characterized by dysfunction and damage to the renal microvasculature^2^. In particular, the glomerulus is the primary site of diabetic injury in the kidney. Glomerular hypertrophy and loss of podocytes, which are involved in glomerular filtration and maintenance of healthy kidney function, have been shown to be hallmarks of progressive DKD, and the degree of podocyte depletion correlates with the stage of the disease^3,4^. In the earlier stages of the disease, glomerular hyperfiltration and hypertrophy, basal glomerular membrane thickening and microalbuminuria are usually observed and are followed by mesangial matrix expansion and proteinuria^5^. Subsequently, nodular glomerulosclerosis and worsening proteinuria develop at an advanced stage, leading to end-stage kidney disease^6^. Glomerular endothelial dysfunction, through the involvement of reactive oxygen species and mitochondrial stress, has been shown to be critical in the early steps of the pathogenesis and progression of DKD^7^. New treatments that aim to restore endothelial function could represent an effective strategy for preventing and treating the early stages of DKD.

In diabetic glomeruli, increased levels of vascular endothelial growth factor (VEGF) leading to new capillary formation and pre-existing capillary elongation have been reported^8^. Furthermore, exacerbated activation of glomerular VEGF signaling in mice has been shown to cause mesangial matrix expansion, which has also been associated with DKD^9^. Therefore, anti-angiogenic therapies targeting VEGF signaling have been explored as options for the prevention and treatment of DKD. Indeed, the renal protective efficacy of anti-VEGF antibodies in diabetic mice have previously been reported^10^. However, concerns have been raised in regards to anti-VEGF unwanted effects on the microcirculation, including potential renal thrombotic microangiopathy^11^. For these reasons, the identification of vascular signaling pathways involved in the pathogenesis of DKD could lead to a better understanding of the molecular events involved in glomerular vascular injury and allow the development of improved therapeutic strategies while minimizing side-effects.

The Bone Morphogenetic Protein (BMP) receptor Activin receptor–like kinase 1 (Alk1), which is predominantly expressed in the vascular endothelium, has been shown to play a critical role in angiogenesis^12–14^. Embryos lacking Alk1 die early during embryonic development due to impaired vascular remodeling and lack of perivascular cell coverage^15^. In renal physiology, Alk1 has been suggested to play an important role in the regulation of extracellular matrix deposition, including collagen type I and fibronectin, and Alk1 heterozygosity has been associated with increased renal fibrosis in a mouse model of obstructive nephropathy, probably due to the decrease in the Alk1/Smad1 antifibrotic/protective signaling in renal fibroblasts^16^.

We have previously shown that chronic hyperglycemia in a model of STZ-induced diabetes in mice leads to impaired Alk1 signaling and contributes to loss of retinal vascular barrier function^17^. Given its critical role in the maintenance of a quiescent, stable endothelium as well as its described effects on perivascular cell recruitment^15,18^, we evaluated the role of Alk1 in the kidney during the development of diabetic nephropathy. In the present study, we show the worsening of kidney function in diabetic mice haploinsufficient for Alk1 solely in the endothelium. Reduced Alk1 expression was associated with microalbuminuria, increased glomerular apoptosis as well as podocyte loss. Together, these data suggest that vascular Alk1 signaling is protective in glomerular endothelial cells in diabetic conditions.

## Material and methods

### Human samples

The study conforms to the tenets of the Declaration of Helsinki, and approval of the human clinical protocol was obtained from the Maisonneuve-Rosemont Hospital Ethics Committee. All subject recruitment procedures and informed consent forms, including consent to use renal biopsy samples for research purposes, were approved by the Maisonneuve-Rosemont Hospital Ethics Committee and written informed consent was obtained from each patient. Renal biopsy specimens with sufficient tissue for immunohistochemical evaluation after the completion of diagnostic workup were included. Demographic and clinical characteristics of patients are shown in Supplementary Table 1.

### Animals

The Maisonneuve-Rosemont Hospital ethics committee, overseen by the Canadian Council for Animal Protection, approved all experimental procedures (protocol number: 2014-18). All the animal experiments were conducted according to the Standard Operation Procedures (SOP) of the Maisonneuve-Rosemont Hospital Animal Ethics Committee. C57BL/6 J mice (Jackson Laboratories) were maintained in the animal research facility in Maisonneuve-Rosemont Hospital. Tamoxifen-inducible Cdh5-CreErt2 and *acvrl1* floxed mice were kindly provided by Ralf Adams^19^ and S. Paul Oh respectively^20^. To generate Alk1ΔEC mice, Cdh5-CreErt2 and *acvrl1* floxed mice were crossed and injected with 50 mg/kg tamoxifen dissolved in corn oil for five consecutive days. Throughout the studies, Cdh5-CreErt2-Alk1^+/+^ (thereafter referred as C5Cre) mice injected with tamoxifen as described above were used as controls.

### Streptozotocin (STZ) induced diabetes

Six to eight-week old male Alk1ΔEC or C5Cre mice were individually marked, weighed and their baseline blood glucose levels determined prior to STZ injection. Mice received daily intraperitoneal (IP) injections of 45 mg/Kg body weight STZ (Sigma-S0130) dissolved in sterile sodium citrate dehydrate buffer, pH 4.5 for 5 consecutive days. For non-diabetic controls, mice received citrate buffer injections. Tail blood glucose was monitored (AlphaTrak 2) every week for up to 8 weeks after the final STZ injection. Mice were considered diabetic if their non-fasted blood glucose levels reached 17 mM (300 mg/dL).

### Urine and blood collection

Urine samples were collected from animals in individual urine collection cages after acclimatization for 24 h and stored at − 80 °C until analysis. Blood was collected from mice through cardiac punctures into tubes containing EDTA for plasma or allowed to clot for 30 min at room temperature for serum, and centrifuged at 4 °C for 15 min at 1 000 × *g*. Serum or plasma were removed and assayed immediately or stored at -80 °C.

### Evaluation of biochemical parameters

Creatinine clearance was used to estimate the glomerular filtration rate (GFR) and was calculated and expressed as milliliters per min per 100 g of body weight^21^. Serum and urine creatinine concentration were determined as previously described (CREP2, Roche Diagnostics, Canada)^22,23^. Briefly, samples were prepared by transferring 50 μl of standard or serum to a 1.5 ml microcentrifuge tube. Proteins were precipitated and supernatant were lyophilized on a speed vac (LABCONCO freeze Dry system, VWR, Canada). Lyophilized samples were reconstituted in 25 μl deionised water and vortex-mixed thoroughly. After a 30 min incubation at room temperature, samples were vortex-mixed thoroughly again and then centrifuged at 11,000 × *g* for 5 min. 8 μl of each supernatant were transferred to a half area plate (Costar #3695), in duplicate. 62 μl of CREP2 R1 buffer was added to each well. The plate was vortex-mixed (MixMate, Eppendorf, Canada) at 1,000 rpm 30 s, and incubated 15 min at 37 °C to allow endogenous creatinine degradation. Readings at 405 nm and 540/630 nm were performed and CREP2 R2 buffer was then added to each well and the plate was vortex-mixed at 900 rpm for 30 s. Readings were performed on a kinetic mode, each minute for a 30 min period (ELx808, BioTek, USA). Urinary Creatinine and urine proteins were measured on an Architect c16000 clinical chemistry analyzer (Abbott Diagnostics, IL, USA), using a kinetic alkaline picrate method and a turbidimetric method respectively. Microalbuminuria was assessed using a Mouse Albumin ELISA Kit (ICL LAB, Portland, OR) according the manufacturer’s instructions.

### Isolation of glomeruli

Mouse kidneys were extracted, minced, and digested in 2 mg/ml collagenase I solution (Gibco) in RPMI-1640 (Invitrogen) at 37 °C for 5 min. Extracts were then filtered through a 70-µm cell strainer and once more through a 40-µm cell strainer. The homogenates were centrifuged at 720 g for 10 min. Isolated glomeruli were then collected in RIPA extraction buffer (20 mM Tris–HCl (pH 7.5), 150 mM NaCl, 1 mM EDTA, 1 mM EGTA 1% NP-40, 1% sodium deoxycholate, 2.5 mM sodium pyrophosphate, 1 mM β-glycerophosphate, 1X Protease inhibitor cocktail (BioBasic)) for protein extraction and processed for immunoblots. Anti-mouse Alk1 (R&D systems), anti-beta actin (Santa Cruz Biotechnology) and peroxidase-labeled secondary antibodies (Vector Laboratories) were used for detection.

### Transmission electron microscopy

Glutaraldehyde-fixed kidney cortical sections were mounted on a copper grid and photographed under a transmission electron microscope (Hitachi H-7500; Tokyo, Japan). Glomerular basement membrane thickness was determined by a blinded observer by calculating the shortest distance between the endothelial cytoplasmic membrane and the outer lining of the lamina rara externa underneath the cytoplasmic membrane of the epithelial foot processes using ImageJ. Assessment of GBM thickness and podocyte foot processes was undertaken on glomerular capillaries (N = 7–10 capillaries/glomeruli) from 3 glomeruli per group. The number of podocyte foot processes per 10 µm glomerular basement membrane was determined in 7–10 glomerular capillaries of each glomeruli, as previously described^24^.

### Determination of glomerular surface area

Kidneys were harvested and fixed in 4% formalin. Paraffin-embedded Sections (5 µm thick) were stained using Haematoxylin/Eosin to evaluate kidney morphology. Glomerular surface area was assessed by a blinded examination of at least 50 glomeruli per Section^25^. Glomerular surface area was measured in captured digital images using ImageJ by tracing around the perimeter of the glomerular capillary tuft using the tracing tool.

### Immunofluorescence

Immunofluorescence was performed using frozen Sections (10 µm). The following antibodies were used as primary antibodies: monoclonal rat anti-CD31 antibody (BD Biosciences); polyclonal rabbit anti-type IV collagen antibody (Abcam); anti-mouse Alk1 antibody (R&D systems); anti-Nephrin antibody (Abcam); anti-WT1 antibody (Abcam); anti-podocin antibody (Abcam); anti-cleaved-caspase 3 (Cell Signaling); anti-PDGFRB (R&D). Alexa Fluor 488 or 647 conjugated antibodies (ThermoFisher Scientific) were used as secondary reagents and slides were mounted with Fluoroshield/DAPI (Sigma). Images were obtained by confocal microscopy (Olympus Fluoview). For quantification of immunofluorescence, staining intensity and area was quantified using 50 randomly selected glomeruli per kidney section. Brightness and contrast were adjusted on displayed images (identically for compared image sets) and quantified (identical threshold settings for compared image sets) using ImageJ. For patient samples, paraffin-embedded tissues were cut into 4- to 6-μm sections and processed for immunofluorescence. Antigen retrieval was performed in citrate solution pH = 6. The sections were then labeled with anti-human Alk1 antibody (R&D systems). Slides were subsequently exposed to specific AF647-conjugated secondary antibody (ThermoFisher).

### Terminal deoxynucleotidyl transferase biotin-dUTP nick end labeling (TUNEL)

Apoptotic nuclei were identified using a TUNEL Assay Kit (Abcam) following the manufacturer’s protocol. Detection was followed by WT1 staining to label podocytes. Apoptotic cells were counted by a blind observer in 35–45 glomeruli per section, using 3 sections per kidney.

### Cell culture

Human Umbilical Vein Endothelial Cells (HUVECs) were obtained from PromoCell and cultured in endothelial growth medium ECGM-2 (Lonza) and kept at 37 °C and 5% CO_2_. For RNAi experiments, cells were seeded in 6-well plate with complete media till 90% confluency was reached. Cells were then transfected with 75 pmol/well of target or control siRNA for 48 h. mRNA was isolated using RNeasy kit (Qiagen) and cDNA was synthesized using iScript cDNA synthesis kit. Primers for quantitative PCR were obtained from QIAGEN (Quantitect primer assays).

### Statistical analysis

All values are expressed as the mean ± standard error (SEM). Statistical analyses were performed using the GraphPad Prism software. Quantitative differences between multiple groups were compared by using one-way ANOVA test, once normality and homogeneity was proved with Shapiro–Wilk test. Non-parametric Mann–Whitney test was used to compare two pair of groups. A level of P < 0.05 was considered statistically significant.

## Results

### Glomerular expression of Alk1 in mice and DKD patients

**Figure 1:**
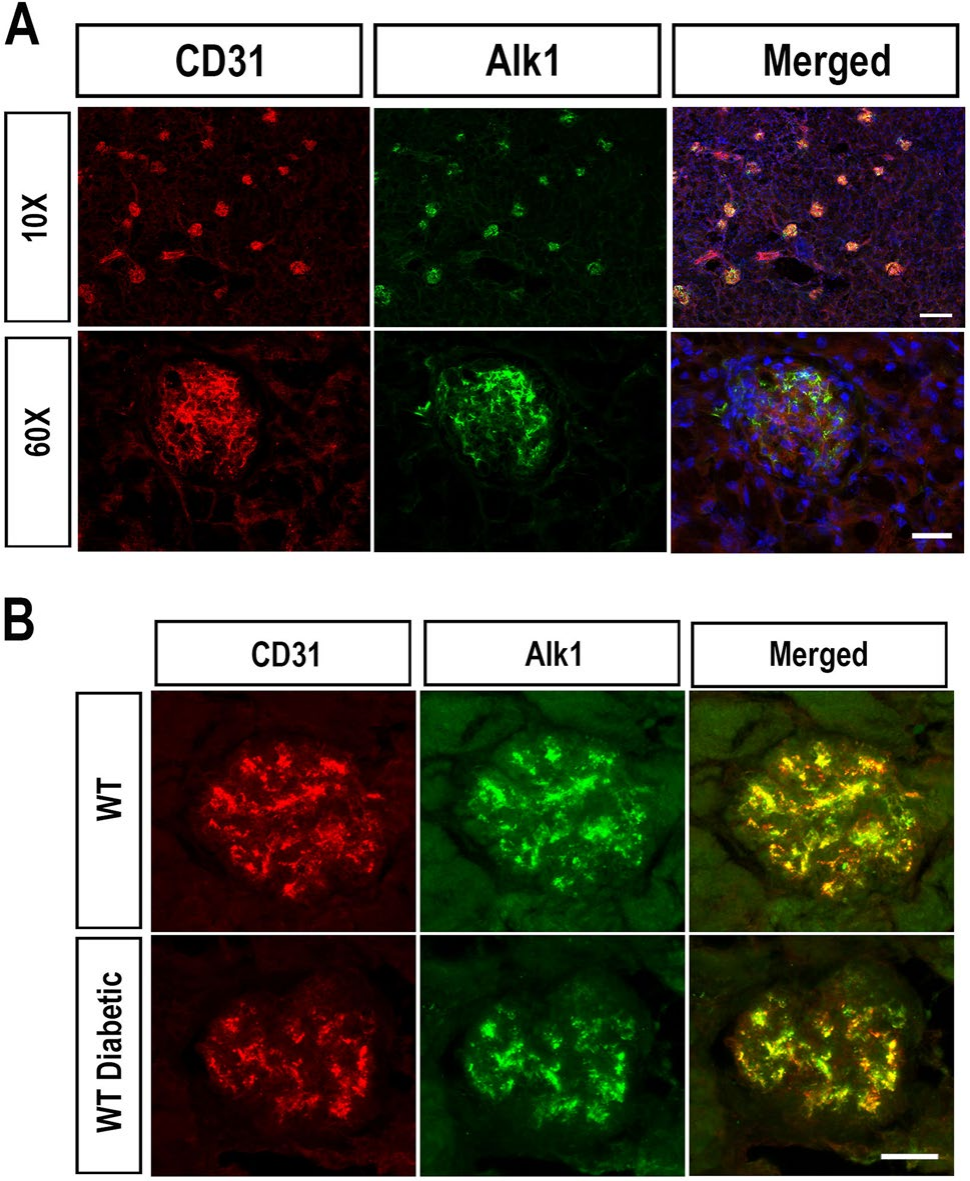
Expression of Alk1 in mouse kidney tissue. (**A**) Immunofluorescent staining of Alk1and CD31 in kidney sections harvested from eight-week old C57/Bl6 mice. Scale Bars: top row: 150 µm; bottom row: 25 µm. (**B**) Expression of Alk1 in C57/Bl6 mice injected eight weeks previously with citrate buffer (top row) or STZ (bottom row). Scale bar: 25 µm.

We have recently shown that chronic hyperglycemia impairs Alk1 signaling in endothelial cells, which in turn impacts retinal barrier function in diabetic animals^17^. Given the importance of the endothelium in glomerular renal filtration and its susceptibility to dysfunction in diabetes, we evaluated the consequences of Alk1 depletion on glomerular endothelial cell function and renal filtration in diabetic animals. We first assessed physiological Alk1 expression in mouse kidney by immunofluorescence. In adult C57BL/6 J mouse kidneys, immunostaining of glomeruli and small blood vessels of the renal interstitium using CD31 and Alk1 antibodies showed that both markers co-localized in the glomerular endothelium (Fig. 1A). No cross-reactivity of either Alk1 or CD31 was observed in the tubular epithelium or interstitial cells. Alk1 expression was also detected in renal biopsies of control subjects, where it was present in endothelial cells of glomeruli, arterioles, interlobular arteries and peritubular capillaries (Supplementary Fig. 1A). Next, as we have previously shown that Alk1 expression is reduced in the pulmonary and retinal endothelium of diabetic mice^17^, we also evaluated Alk1 expression in the glomerular endothelium in C57BL/6 J mice eight weeks after the onset of STZ-induced diabetes and in renal biopsies obtained from diabetic nephropathy patients. In control diabetic mice, Alk1 expression, while showing a slight decrease, was not significantly reduced in glomeruli eight weeks after the onset of diabetes (Fig. 1B). However, histological assessment of Alk1 expression in the glomeruli of patients with diabetic nephropathy revealed a significant loss of Alk1 glomerular immunostaining compared with non-diabetic patients (Supplementary Table 1; Supplementary Fig. 1B). The observation that Alk1 was down-regulated in diabetic patients, which were characterized with long-term chronic kidney disease, and not in diabetic mice may be reflective of the advanced stage of diabetic nephropathy.

### Evidence of urine albumin excretion and hyperfiltration in Alk1-deficient diabetic mice

**Figure 2:**
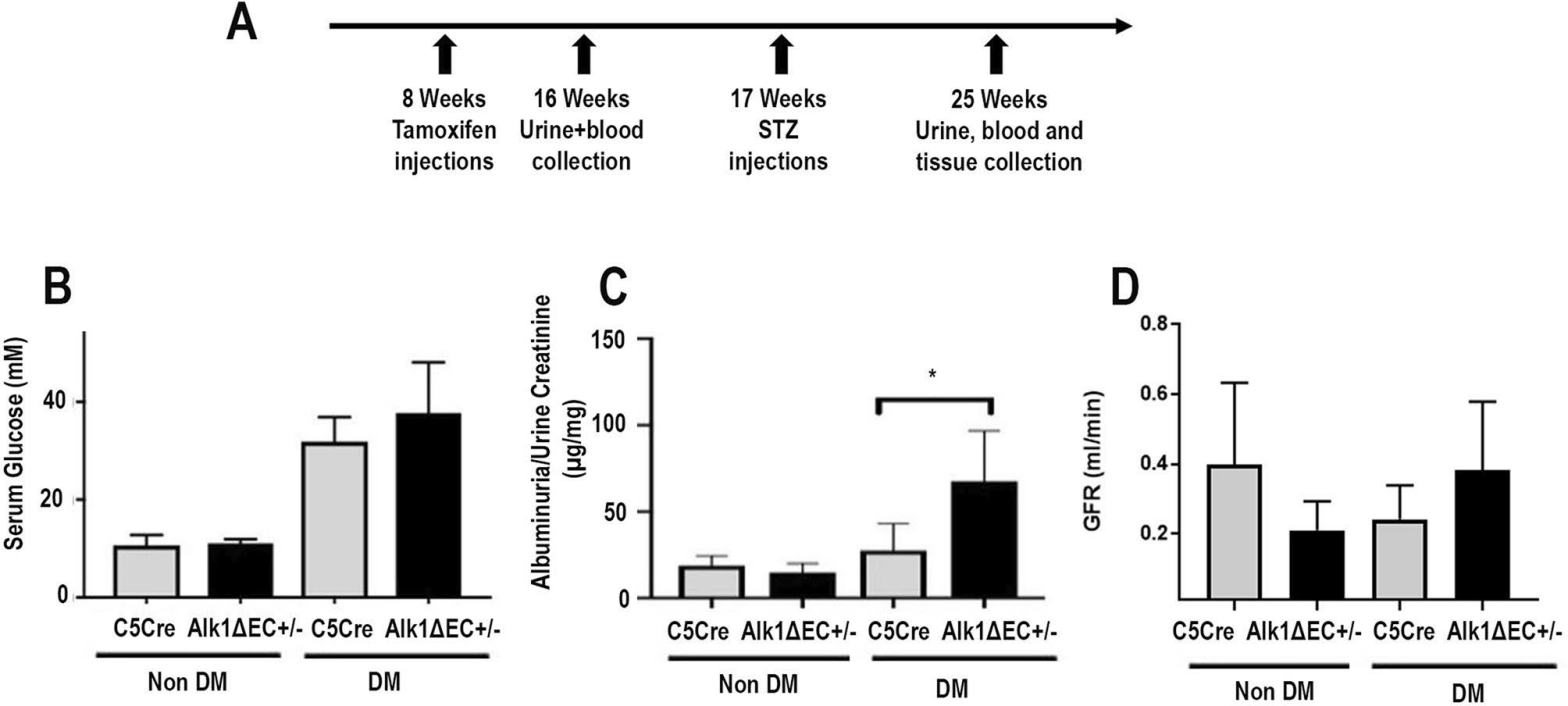
Evaluation of albuminuria in Alk1ΔEC +/− diabetic mice. (**A**) Experimental time line for the generation of diabetic Alk1ΔEC +/− mice, in which CRE-Ert2 expression was placed under the control of the VE-Cadherin promoter (Cdh5-CRE). For inactivation of Alk1 in the endothelium, Cdh5-CRE-Ert2 (C5Cre) transgenic mice were crossed with homozygous or heterozygous acvrl1 floxed allele mice. (**B**) Measurement of blood glucose levels in C5Cre and Alk1ΔEC +/− eight weeks after injection of sodium citrate or STZ. (**C**) Measurement of ratio of albumin to creatinine levels in C5Cre and Alk1ΔEC +/− eight weeks after injection of sodium citrate or STZ. (**D**) Measurement of glomerular filtration rate in C5Cre and Alk1ΔEC +/− eight weeks after injection of sodium citrate or STZ. *p < 0.05, (n = 8 mice/group).

In order to characterize the consequences of Alk1 deletion in the endothelium of diabetic mice on renal function, we used mice bred on a C57/Bl6 background with conditional deletion of *acvrl1* in the endothelium (Alk1ΔEC)^17^. The C57/Bl6 strain has been reported to be relatively resistant to the development of renal injury in experimental models of kidney diseases, including DKD^26^. Indeed, albuminuria and renal pathological changes are less commonly observed in diabetic C57/Bl6 mice than in other strains. However, several specific genetically modified mice with a C57/Bl6 background were shown to progress to diabetic nephropathy when diabetically-induced, such as the eNOS knockout in STZ-induced diabetic or db/db mice and the ApoE knockout in STZ-induced mice^27^. Given that Alk1 deficiency has been associated with eNOS uncoupling, reduced NO, and increased reactive oxygen species (ROS) production^28^, we evaluated the consequences of Alk1 deficiency on renal function and whether diabetic Alk1 haploinsufficient mice would be more susceptible to develop renal dysfunction. To address this, C5Cre and heterozygous Alk1ΔEC mice, in which Alk1 is specifically deleted in the endothelium, received tamoxifen followed eight weeks later by STZ injections to induce diabetes (Fig. 2A). Heterozygous Alk1ΔEC mice were used for STZ diabetes experiments, as the severity of the vascular phenotype and the short-term lethality of homozygous Alk1ΔEC mice^29^ preclude long-term experiments. While heterozygous Alk1ΔEC mice develop no detectable blood vascular malformations, we have recently demonstrated that chronic hyperglycemia leads to impaired Alk1 signaling and vascular permeability defects in these animals^17^. Following STZ or citrate injections, blood glucose levels were measured weekly (Supplementary Fig. 2A) and all mice were euthanized 8 weeks later. The induction of diabetes in STZ-injected mice was confirmed by measuring blood glucose levels, which were not significantly different between C5Cre (33 mM) and Alk1ΔEC (37 mM) mice (Supplementary Fig. 2A,B). Decreased Alk1 expression in glomeruli of Alk1ΔEC +/− diabetic mice was shown by immunofluorescence and by western blot analysis of isolated glomeruli eight weeks after the induction of diabetes (Supplementary Fig. 1B,C). Eight weeks after STZ or citrate injections, mice were monitored for renal function by measuring creatinine clearance, microalbuminuria and urine electrolytes. In non-diabetic animals, Alk1 haploinsufficiency had no significant effects on urinary albumin excretion, body weight, daily urine volume, urine and serum electrolytes and serum creatinine (data not shown). Additionally, the presence of albuminuria, as assessed by the albumin-to-creatinine ratio of 24-h urine albumin excretion at 8 weeks post-diabetes, was not observed in C5Cre diabetic mice (Fig. 2C), which is consistent with studies showing that mice bred on a C57/Bl6 background are refractory to diabetes-induced kidney disease^26^. However, in contrast to C5Cre mice, Alk1ΔEC diabetic mice displayed microalbuminuria, an early sign of DKD (Fig. 2C). Significantly, no changes in glomerular filtration rate (Fig. 2D) or urine electrolytes were observed (Supplementary Fig. 3) between C5Cre and Alk1ΔEC diabetic mice, suggesting that Alk1 haploinsufficiency does not affect reabsorption in proximal tubules. Taken together, these data suggest that reduced Alk1 expression in the glomerular endothelium may predispose the kidney to glomerular dysfunction rather than affecting tubule function in diabetic conditions.

### Glomerular alterations in Alk1-deficient diabetic mice

**Figure 3:**
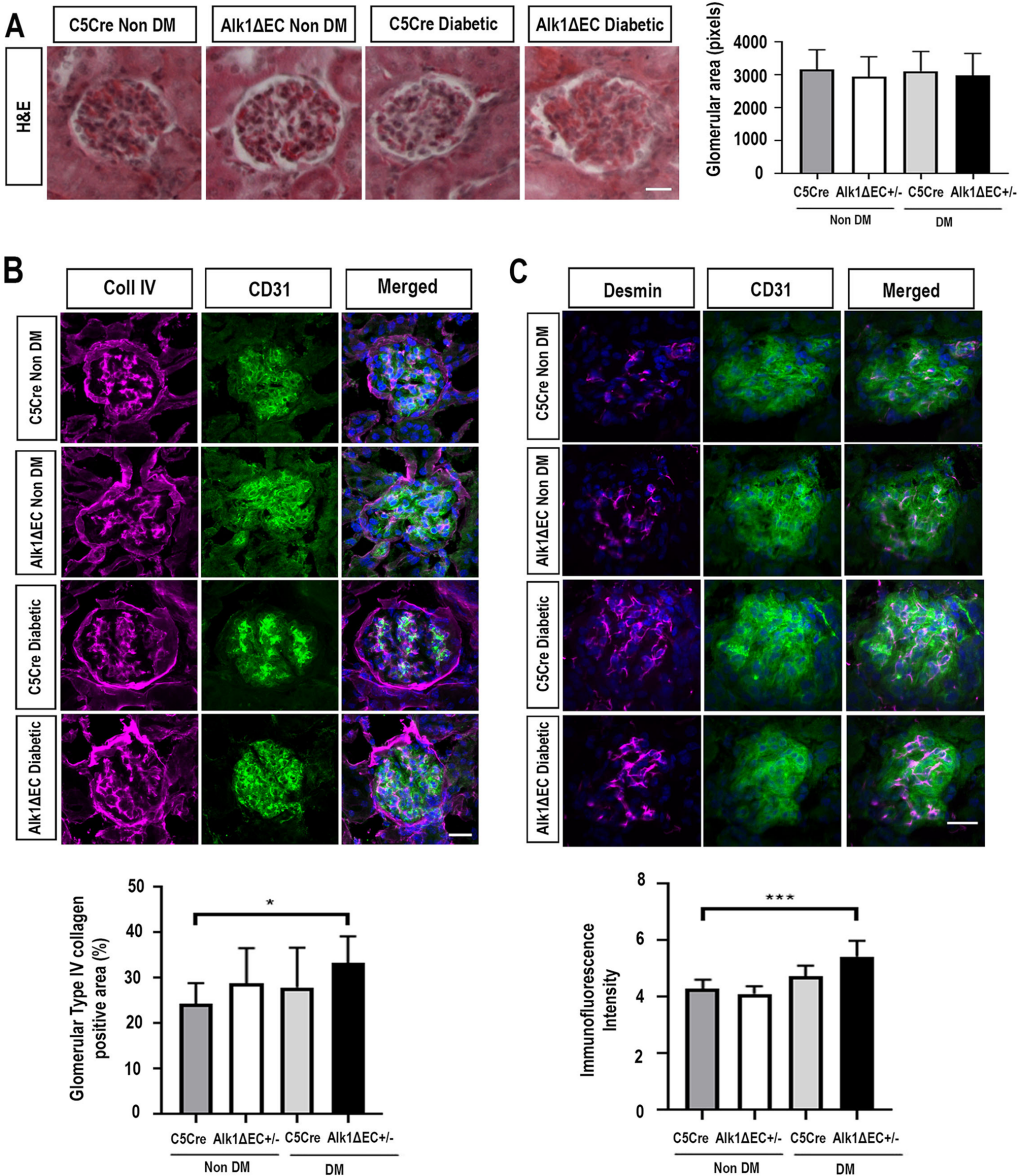
Alk1 haploinsufficiency exacerbates collagen IV matrix accumulation and podocyte injury indicator desmin in diabetic mice. (**A**) Glomerular area analysis in non-diabetic C5Cre and diabetic C5Cre and Alk1ΔEC +/− mice eight weeks after the onset of diabetes. Images show representative H&E, along with quantification of 50 glomeruli from 8 to 10 mice. (**B**) The glomerular accumulation of type IV collagen was assessed by immunofluorescence for non-diabetic or diabetic C5Cre and diabetic Alk1ΔEC +/− mice. Immunoreactivity for type IV collagen was enhanced in diabetic Alk1ΔEC +/− mice compared with non-diabetic C5Cre mice. (**C**) Immunofluorescence analysis of desmin and quantification in non-diabetic or diabetic C5Cre and diabetic Alk1ΔEC +/− mice. Analysis show quantification of at least 50 glomeruli from 5 mice/group. *p < 0.05, **p < 0.01. Scale bars: 25 µm.

As our data suggest changes in glomerular barrier function in diabetic Alk1-deficient mice, we evaluated glomerular histology in C5Cre and Alk1ΔEC non-diabetic and diabetic mice. Diabetes-induced glomerular hypertrophy is an early sign of renal dysfunction in diabetic patients^17^. However, no significant changes in glomerular size could be observed between diabetic C5Cre and Alk1-haploinsufficient diabetic mice by fluorescence microscopy (Fig. 3A). Loss of glycocalyx, a complex set of varied EC membrane–associated macromolecules on the endothelial cell surface layer, has been associated with albuminuria in diabetic nephropathy. To examine whether chronic hyperglycemia combined with Alk1 haploinsufficiency is associated with damage of the glomerular endothelial surface layer, kidney cryostat sections taken from mice eight weeks after the onset of diabetes were stained with wheat germ agglutinin (WGA), a lectin that binds to negatively charged sugar residues of glycoproteins, such as sialic acid, and with heparanase, an endo-beta-D-glucuronidase that specifically cleaves the heparan sulfate chain of negatively-charged proteoglycans. However, no associations with a reduction in glycocalyx staining, as measured using lectin or heparanase staining, was observed (Supplementary Fig. 4). While studies suggest that diabetes-induced glycocalyx alterations as a significant cause of increased albumin excretion, our data may suggest that other diabetes-related factors, for example podocyte dysfunction or glomerular basement membrane alterations might better explain how Alk1 depletion leads to increased albumin excretion during development of diabetic nephropathy^30,31^.

Glomerular accumulation of mesangial matrix, including type IV collagen is an early hallmark of DKD^32^. Eight weeks after the onset of diabetes, no significant changes in collagen IV deposition were observed between C5Cre diabetic and non-diabetic animals nor between C5Cre and Alk1ΔEC non-diabetic mice (Fig. 3B). However, compared to diabetic C5Cre mice, Alk1ΔEC diabetic mice showed significant increase in glomerular deposition of type IV collagen (Fig. 3B). These data are in accordance with previous studies showing that Alk1 heterozygocity is associated with increased matrix deposition by mesangial fibroblasts^16,33^. Furthermore, quantitative PCR analysis of ACVRL1 siRNA-transfected endothelial cells, also showed a significant increase in α1 type IV collagen (COL4A1) expression compared to controls (Supplementary Fig. 5). Other glomerular basement membrane molecules, including fibronectin, Laminin α4, Laminin β2 or Laminin γ1 did not change, while Laminin β1 levels were decreased following Alk1 downregulation. We subsequently investigated podocyte injury by evaluating the expression of desmin, a biomarker of injured podocytes. Our data show an increase in desmin-positive cells in diabetic Alk1-deficient mice compared to non-diabetic C5Cre mice (Fig. 3C). Together, these data suggest that Alk1 deficiency may accelerate glomerular extracellular matrix production and glomerular damage in diabetic mice.

**Figure 4:**
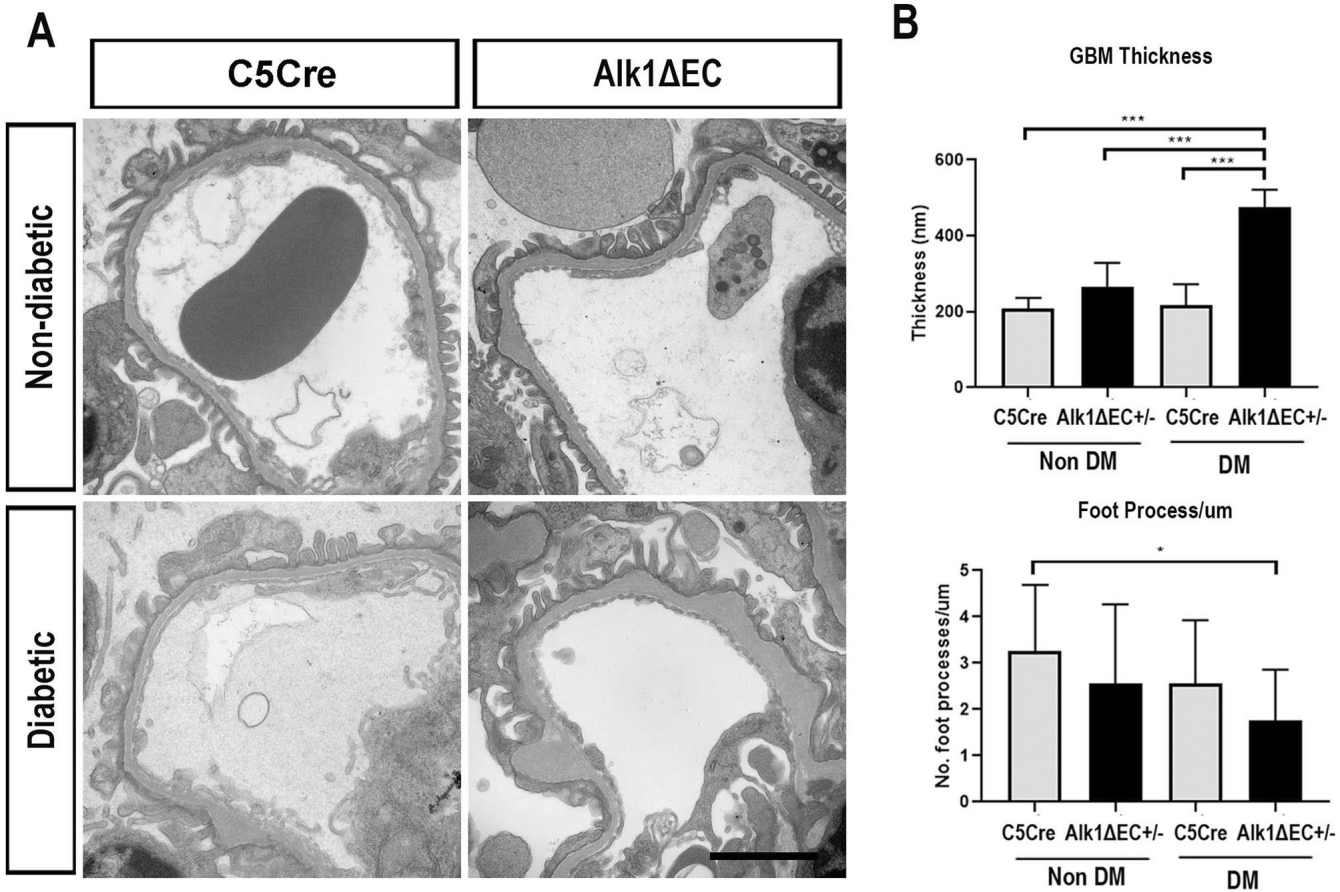
Increased glomerular basement membrane thickness in diabetic Alk1ΔEC +/− mice. (**A**) Representative glomerular electron micrographs from non-diabetic or diabetic C5Cre or Alk1ΔEC +/− mice. (**B**) Diabetic Alk1ΔEC +/− glomeruli developed an increased GBM width and decreased podocyte foot processes per μm length compared to diabetic C5Cre glomeruli (*p < 0.05). (n = 3 mice). Scale bar: 2 µm.

Cross-talk amongst podocytes, endothelial cells and the basement membrane is essential for the maintenance of the glomerular filtration barrier. We evaluated the consequences of Alk1 deletion in endothelial cells on the glomerular basement membrane. Transmission electron microscopy showed that, while there was no difference in slit membrane structure between C5Cre and Alk1ΔEC mice under non-diabetic conditions (upper panels of Fig. 4A), Alk1ΔEC diabetic mice displayed significant thickening of the glomerular basement membrane compared to diabetic C5Cre mice (lower panels of Fig. 4A). In addition, while there were no differences in foot processes between non-diabetic and diabetic C5Cre mice, an overall decrease in the number of foot processes was observed in diabetic Alk1ΔEC mice (Fig. 4B), suggesting that loss of endothelial Alk1 can predispose to glomerular structural changes and podocyte alterations in diabetic animals.

**Figure 5:**
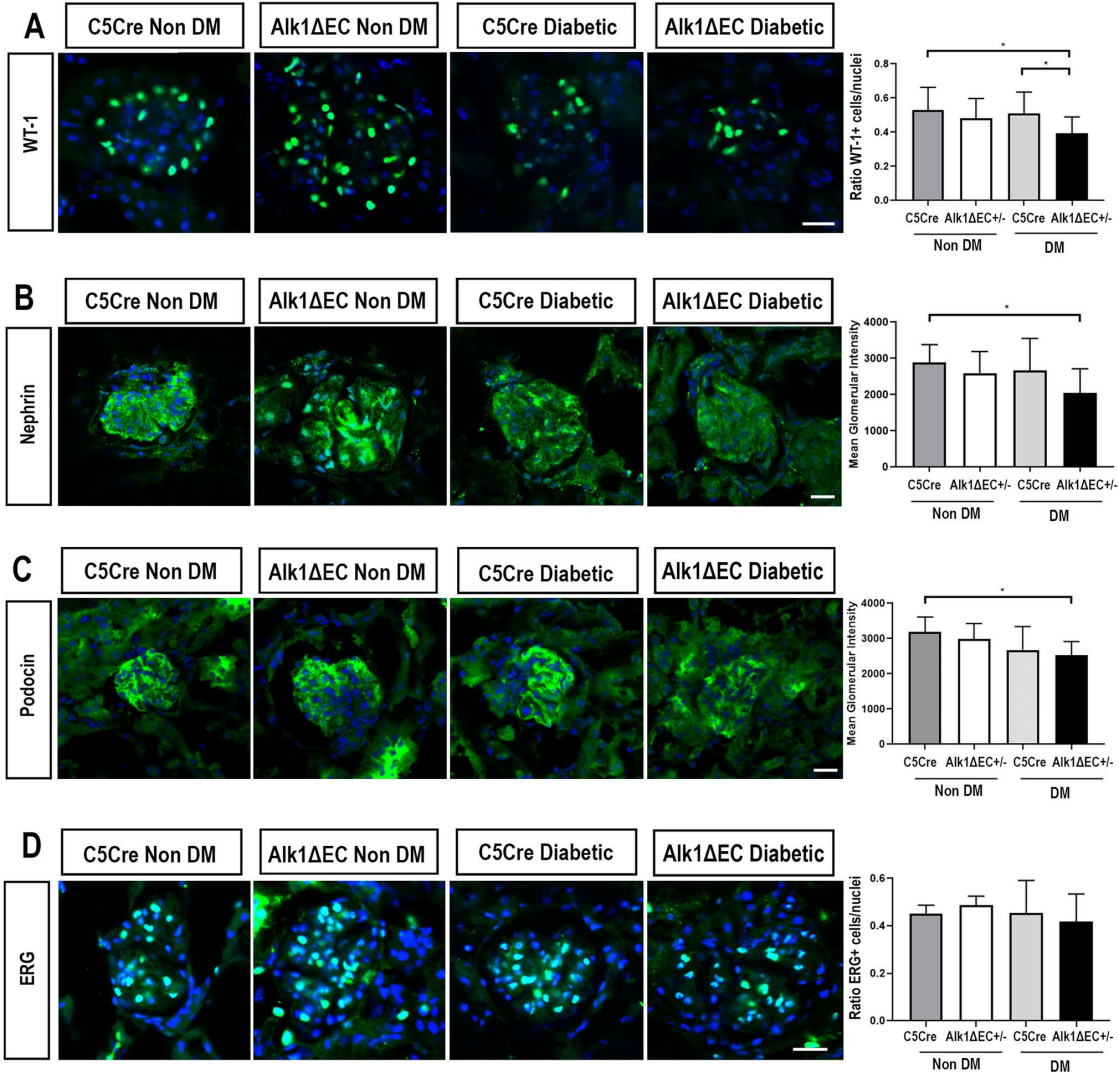
Effects of Alk1 haploinsufficiency on podocytes and endothelial cells in non-diabetic or diabetic C5Cre and diabetic Alk1ΔEC +/− mice Immunofluorescence analysis of WT1 (**A**), nephrin (**B**), podocin (**C**) and ERG (**D**). Quantification was performed by evaluating the number of positive cells per glomerular nuclei for WT1 and ERG staining, or by assessing glomerular staining intensity for nephrin and podocin. Analysis show quantification of at least 50 glomeruli from 5 mice/group. *p < 0.05. Scale bars: 25 µm.

Using WT1 staining, which stains the nuclei of glomerular podocytes, we observed that Alk1 conditional deletion in endothelial cells resulted in significant podocyte loss in diabetic mice (Fig. 5A). The decrease of WT1-expressing cells in Alk1ΔEC diabetic mice was also accompanied by a reduction in the expression of nephrin, which encodes a member of the immunoglobulin family of cell adhesion molecules implicated in the glomerular filtration barrier in the kidney (Fig. 5B). Furthermore, a similar reduction was observed in podocin, a membrane protein that oligomerizes in lipid rafts together with nephrin to form filtration slits (Fig. 5C). To evaluate the consequences of Alk1 deficiency in glomerular endothelial cells, ERG staining, which specifically labels endothelial nuclei, was performed. However, we did not observe significant losses of ERG-positive endothelial cells between non-diabetic and diabetic C5Cre and Alk1ΔEC diabetic mice (Fig. 5D). Together, these data suggest that Alk1 deletion in diabetic glomerular endothelial cells causes glomerular injury through the loss of podocytes rather than endothelial cells.

**Figure 6:**
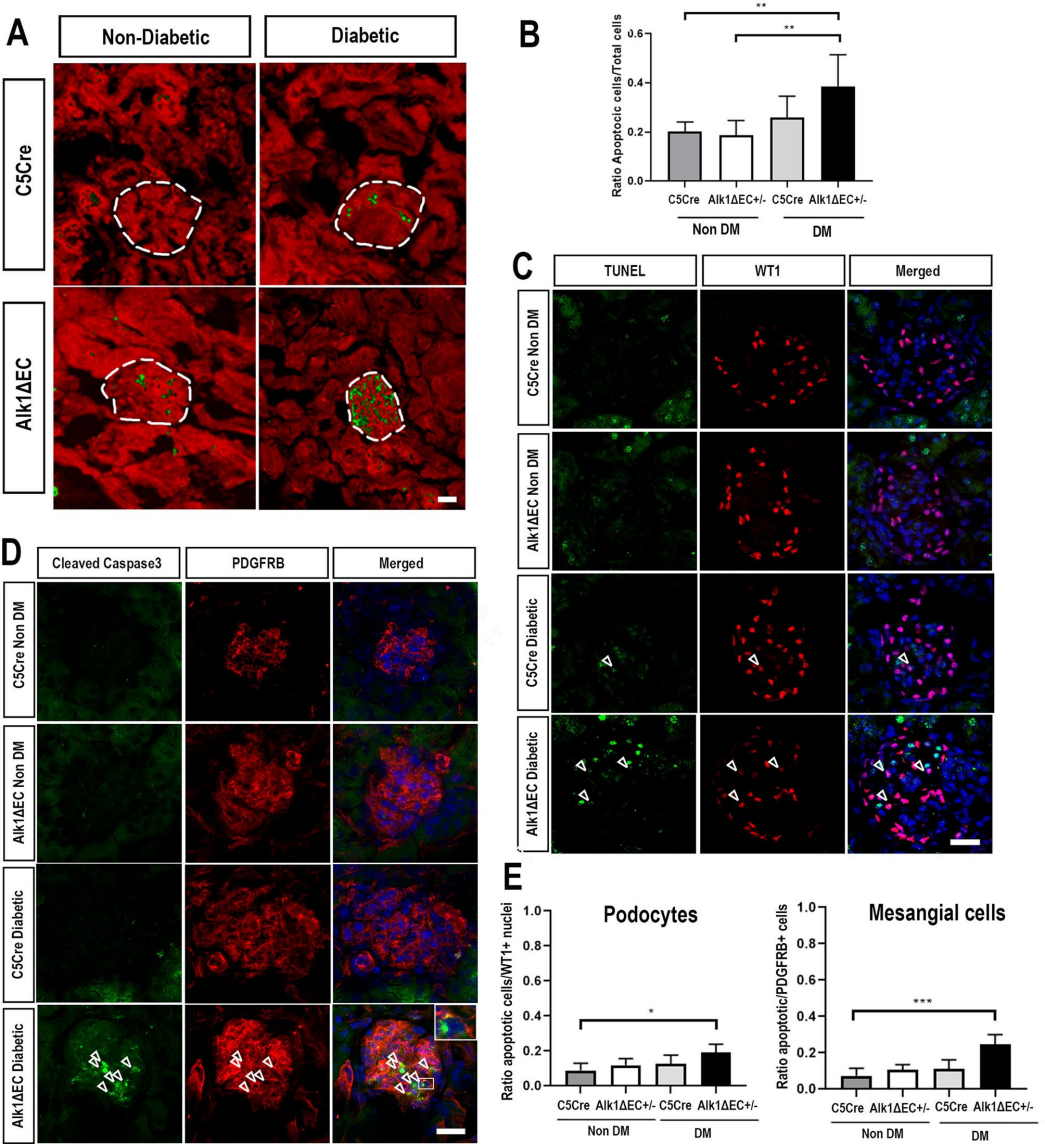
Increased number of glomerular apoptotic cells in diabetic Alk1ΔEC +/− mice. (**A**) Immunofluorescence analysis of cleaved caspase 3 (green) in non-diabetic or diabetic C5Cre and diabetic Alk1ΔEC +/− mice. (**B**) Quantification was performed by evaluating the number of positive cells per glomerular nuclei. (**C**) TUNEL and WT1 immunofluorescent staining of non-diabetic or diabetic C5Cre or Alk1ΔEC +/− mice. (**D**) Cleaved-caspase 3 and PDGFRB immunostaining of non-diabetic or diabetic C5Cre and Alk1ΔEC +/− mice. (**E**) Quantification was performed by evaluating the number of double-positive cells per glomerular nuclei. Analysis show quantification of at least 50 glomeruli from 5 mice/group. **p < 0.01. Scale bars: 60 µm.

To quantify the rate of glomerular apoptosis, cleaved caspase 3 staining was performed (Fig. 6). Positive cells in 50 glomeruli of at least five animals of each group were counted. No significant changes in glomerular apoptosis was detected in C5Cre mice, both in non-diabetic or diabetic conditions. However, the number of glomerular cells undergoing apoptosis was significantly increased in Alk1ΔEC diabetic mice compared to non-diabetic C5Cre or Alk1ΔEC animals (Fig. 6A,B). It is noteworthy that apoptosis appeared to be restricted mostly to the glomeruli. To identify which glomerular cell type underwent apoptosis, double labeling of TUNEL-positive nuclei with WT1 was performed. Surprisingly, we observed that while there was a small increase in podocyte apoptosis (Fig. 6C,E), the majority of apoptotic glomerular cells were not podocytes. Instead, double labeling of cleaved caspase 3 with PDGFRB showed that most glomerular apoptotic cells were mesangial cells (Fig. 6D,E). As a multidirectional cross-talk between podocytes, mesangial cells and endothelial cells is required for the proper function of the glomerulus, our data suggest that disruption of Alk1 signaling in hyperglycemic endothelial cells can cause thickening of the glomerular basement membrane and adversely affect how endothelial cells communicate with non-vascular glomerular cells and ultimately lead to podocyte and mesangial cell dysfunction and apoptosis.

### Alk1 homozygosity leads to glomerular apoptosis

**Figure 7:**
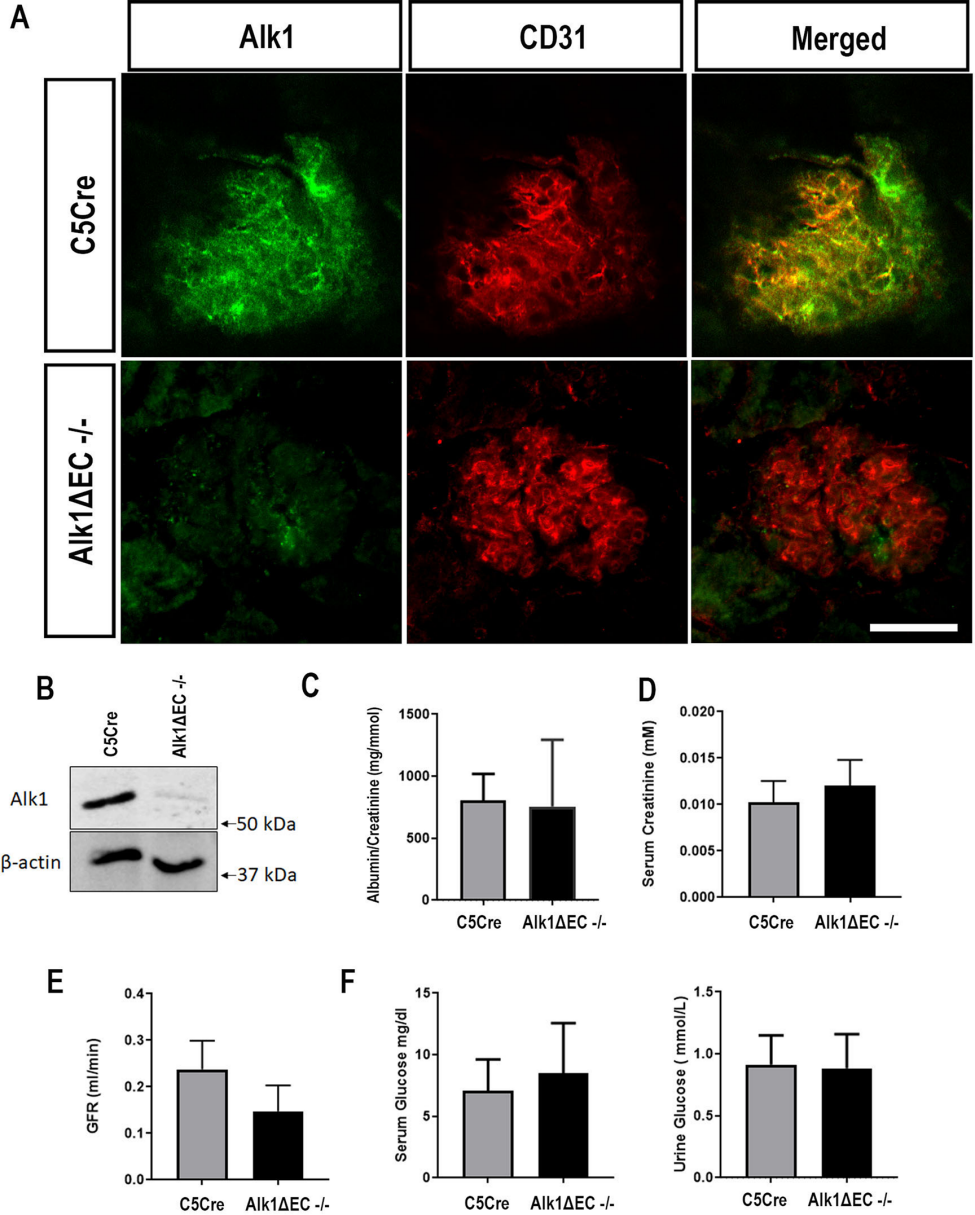
Effects of Alk1ΔEC homozygote deletion on renal function. (**A**) Immunofluorescent staining of Alk1 in kidney sections of C5Cre and Alk1ΔEC−/− mice seven days after tamoxifen delivery. (**B**) Immunoblot of Alk1 expression in isolated glomeruli of C5Cre and Alk1ΔEC−/− mice after tamoxifen injections. Urine albumin (**C**), serum creatinine (**D**), glomerular filtration rate (**E**), serum and urine glucose (**F**) in C5Cre and Alk1ΔEC−/− mice 7 days following tamoxifen injections. (n = 8 mice/group). Scale bar: 50 µm.

We have previously shown that chronic hyperglycemia results in impaired Alk1 signaling^17^. The effects observed in heterozygous Alk1ΔEC diabetic mice may therefore be a combination of Alk1 haploinsufficiency which, combined with hyperglycemia, further inhibits Alk1 signaling leading to glomerular endothelial cell dysfunction. To evaluate whether complete loss of Alk1 was sufficient to induce glomerular dysfunction, we evaluated glomerular filtration in non-diabetic homozygote Alk1ΔEC mice. As homozygote Alk1ΔEC mice display high lethality after tamoxifen injections, we evaluated renal function 7 days after the onset of Alk1 deletion. Loss of Alk1 expression in glomeruli of homozygote Alk1ΔEC mice was confirmed by immunofluorescence (Fig. 7A) and by immunoblotting performed on isolated glomeruli (Fig. 7B). Five days following tamoxifen-induced deletion of Alk1, mice were placed in collection cages and serum and 24 h urine were collected. C5Cre mice injected with tamoxifen were used as controls. Contrary to what was observed in diabetic heterozygote Alk1ΔEC mice, no albuminuria was observed in non-diabetic homozygote Alk1ΔEC mice (Fig. 7C). Furthermore, no significant changes were detected in serum creatinine (Fig. 7D), glomerular filtration rate (Fig. 7E) urine or serum glucose (Fig. 7F). Serum and urine electrolytes levels were also measured in C5Cre and Alk1ΔEC−/− mice (Supplementary Fig. 6), and showed decreased levels of urine sodium and chloride levels in Alk1ΔEC−/− mice. Since urine albuminuria and serum electrolytes did not vary between C5Cre and Alk1ΔEC−-/− mice, changes in sodium and chloride levels in urine may be indicative of vascular hyperpermeability or early cardiac failure, which have been associated with Alk1 loss-of-function^17,34–36^.

**Figure 8:**
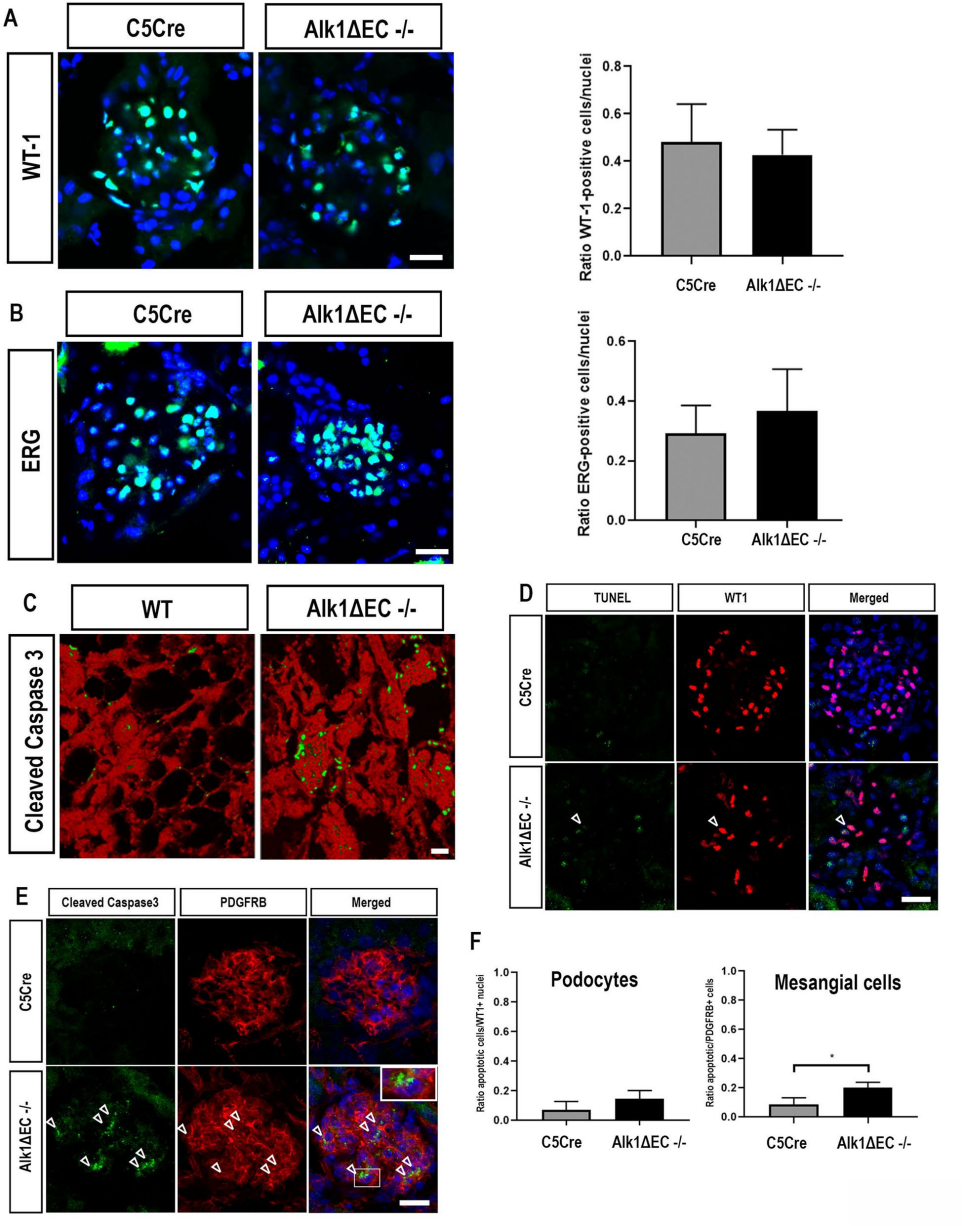
Effects of Alk1 homozygous deletion on podocytes and endothelial cells in C5Cre and Alk1ΔEC−/− mice. Immunofluorescence analysis of WT1 (**A**), ERG (**B**), and cleaved caspase-3 (**C**). (**D**) TUNEL and WT1 immunofluorescent staining of non-diabetic C5Cre and Alk1ΔEC−/− mice. (**E**) Cleaved-caspase 3 and PDGFRB immunostaining of non-diabetic C5Cre or Alk1ΔEC−/− mice. (**F**) Quantification was performed by evaluating the number of double-positive cells per glomerular nuclei. Analysis show quantification of at least 50 glomeruli from 5 mice/group. *p < 0.05. Scale bars: 25 µm.

Even though complete Alk1 deletion did not result in glomerular filtration defects within the time-frame of these experiments, we evaluated whether it could result in early glomerular alterations which could predispose to the development of glomerular dysfunction. While immunofluorescence staining did not reveal changes in WT1-positive glomerular podocytes (Fig. 8A) or ERG-positive endothelial cells in homozygote Alk1ΔEC−/− mice compared to C5Cre mice (Fig. 8B), we did observe increased number of apoptotic cells in the glomeruli of Alk1ΔEC−/− mice compared with C5Cre mice, which may be a sign of early glomerular damage (Fig. 8C). Double immunolabeling of apoptotic cells with WT1 (Fig. 8D) or PDGFRB (Fig. 8E) showed no changes in the number of apoptotic podocytes but revealed that most of the apoptotic glomerular cells were mesangial cells (Fig. 8F). Taken together, these data suggest that lack of Alk1 signaling can lead to early glomerular apoptosis, which may precede podocyte loss. While we did not observe microalbuminuria in Alk1ΔEC−/− mice, the lack of effects on glomerular filtration may be resultant from the relative short time-frame of the experimental settings.

## Discussion

Global endothelial dysfunction has been closely associated with diabetes. Some evidence suggests that endothelial dysfunction is present in the early stages of renal insufficiency and that it plays a role in the progression of renal disease. Thus, the glomerular endothelium, which has been linked to the development of proteinuria as a hallmark of diabetic nephropathy, may be a prominent target for damage in longstanding diabetes. Indeed, gene expression analysis of experimental DKD, as well as biopsies of patients with advanced diabetic nephropathy, has shown significant changes in endothelial gene expression consistent with glomerular endothelial cell dysfunction. Specifically, several genes of the TGF-β family, which play a prominent role in renal cell hypertrophy and extracellular matrix accumulation, have been shown to be dysregulated in the diabetic glomerular endothelium^37^. For example, TGF-β1 expression has been found to be increased in diabetic mice presenting glomerulosclerosis and albuminuria, and contributes to disease progression through its effects on extracellular matrix deposition, including collagen IV, fibrosis and angiogenesis^38,39^. Dysregulation of other factors involved in TGF-β signaling, including BAMBI^40^, TGFBR2 and TGFBR3^41^ have also been shown to be predisposing factors for the development of nephropathy in diabetic individuals.

Alk1 is a vascular-specific receptor of the TGF-β family and binds the circulating factors BMP9 and BMP10^42,43^. Extensive work has been performed in the vascular field on Alk1, demonstrating its capacity to regulate morphogenesis, and its role as a quiescence receptor for endothelial cells^42^. Alk1 signaling is critical for the development of mature, functional vessels, as is made evident by Alk1-deficient homozygous mice displaying severe vascular developmental defects, including fusion of major arteries and veins, impaired placental vascular development and deficient perivascular cell coverage, and consequently die by mid-gestation^15^. In adults, Alk1 helps prevent the formation of vascular lesions and plays a crucial role in endothelial quiescence, as demonstrated by the inducible-deletion of Alk1 in the endothelium at the post-natal stage, which results in rapid lethality^17,29^.

We have recently shown that in a model of STZ-induced diabetes, there is a significant impairment of Alk1 signaling in the retinal endothelium^17^. Inhibition of Alk1 signaling in hyperglycemic endothelial cells resulted in the destabilization of vascular junctions, leading to fluid and protein leakage in the diabetic retina. Given the critical role of Alk1 in the maintenance of a mature, functional endothelium, we evaluated how long-term hyperglycemia affects Alk1 signaling, and the consequences of Alk1 haploinsufficiency on renal function. Under diabetic conditions, we revealed the worsening of urine albumin excretion and glomerular alterations within diabetic mice that were Alk1-haplodeficient in kidney endothelial cells. The data we collected provide evidence that genetic elimination of Alk1 renders mice on a C57BL/6 background more susceptible to the development of diabetic glomerular abnormalities, as determined by proteinuria and increased glomerular extracellular matrix production. The observation that non-diabetic Alk1ΔEC haploinsufficient mice did not present urine albumin excretion and glomerular alterations suggest that an additional detrimental signal, such as chronic high glucose levels, is necessary to trigger vascular dysfunction in endothelial cells haploinsufficient for Alk1. Because Alk1 expression is also downregulated in glomeruli from biopsies of patients with diabetic nephropathy, these considerations may also apply to human diabetic glomerulopathy, where therapeutic intervention using strategies to modulate BMP signaling may be of therapeutic interest.

Some of the main hallmarks of the inhibition of Alk1 signaling in the diabetic glomerular endothelium were the increased deposition of collagen IV basement membrane and the significant loss of podocytes. Collagen IV basement membrane thickness increased significantly in diabetic Alk1ΔEC mice. These changes are consistent with a previous study showing that heterozygous Alk1 expression increased expression of ECM proteins in fibroblasts due to an alteration of TGF-β signaling, leading to an increase in Smad2 and Smad3 phosphorylation^33^. Our data show that Alk1 expression in the endothelium can also play an important role in building and maintaining the glomerular basement membrane and that its inhibition can lead to increased thickness and alter its composition by increasing type IV collagen production in the glomerular endothelium, which has been associated with the progression of diabetic nephropathy^44^.

The loss of podocytes in Alk1ΔEC diabetic mice was associated with increased albuminuria. The reason for the increased proteinuria and decreased podocyte numbers in diabetic Alk1ΔEC mice is unclear but could be due to altered endothelial and podocyte cross-talk. Indeed, in the vascular endothelium, Alk1 signaling has been shown to play a role in the recruitment and function of perivascular cells such as pericytes, which occurs in part through the regulation of factors such as PDGF-B and Jagged1 by Alk1 signaling, which are essential in endothelial/perivascular cell interactions^13,18^. Therefore, heterozygous loss of Alk1, combined with chronic hyperglycemia and changes in basement membrane composition, could lead to the destabilization of endothelial/podocyte cross-talk, which could accentuate podocyte apoptosis and result in glomerular filtration defects. This is in agreement with previous studies showing that disruption of glomerular endothelial cells can lead to podocyte apoptosis^45,46^. While a significant increase in WT1 + podocyte apoptosis was observed in diabetic Alk1ΔEC, the majority of apoptotic cells were PDGFRB + mesangial cells. While it is unclear how Alk1 deletion in the endothelium results in mesangial cell apoptosis, the importance of the endothelium in mesangial cell function is well described^47^. Indeed, it is likely that Alk1 inhibition alters the expression of several paracrine factors involved in mesangial cell survival. One such paracrine factor may be Angiopoietin-2 (Angpt2), which has been shown to induce mesangial cell apoptosis under hyperglycemia^48^. Several studies have indeed shown that Alk1 inhibition does result in increased levels of Angpt2^49,50^. In contrast to diabetic Alk1ΔEC haploinsufficient mice, we did not observe significant podocyte loss in non-diabetic Alk1ΔEC−/− mice, as experiments could only be performed over 7 days due to the lethality of these mice. However, we did observe a significant increase in mesangial cell apoptosis, which may be precursor to podocyte loss.

Overall, our results point to a role for Alk1 in protecting the glomerular endothelium during chronic hyperglycemia. Alk1 is downmodulated in a model of diabetes in mice and in biopsies from patients with established diabetic nephropathy. Decreased Alk1 signaling in diabetes may be an early step in the development of glomerular endothelial dysfunction, leading to podocyte loss. This novel mechanism of Alk1 action in the glomerulus may be of significance not only for diabetic proteinuria but also for other diseases leading to glomerulosclerosis.

## Supplementary information

Supplementary file1 (PDF 1032 kb)
