# Supplementary material for: Alk1 haploinsufficiency causes glomerular dysfunction and microalbuminuria in diabetic mice
Source: Sci Rep. 2020 Aug 4;10:13136. doi: 10.1038/s41598-020-68515-z (PMC7403732; doi:10.1038/s41598-020-68515-z)
Supplement: Supplementary file 1 — Supplementary file1 (PDF 1032 kb) [file 41598_2020_68515_MOESM1_ESM.pdf]

Alk1 haploinsufficiency causes glomerular dysfunction and microalbuminuria in diabetic mice

Cindy Lora Gil, Nathalie Henley, François A. Leblond, Naoufal Akla, Louis-Philippe Laurin, Virginie Royal, Casimiro Gerarduzzi, Vincent Pichette and Bruno Larrivée

Supplementary Information

Participant	Age	Sex	eGFR (ml/min/1,73m^2)	Serum Creatinin (µmol/L)
**Diabetic Nephropathy**				
**1**	65	M	20	146
**2**	76	F	38	118
**3**	48	M	8	667
**4**	79	M	29	208
**5**	79	F	16	284
**6**	71	F	42	112
**7**	66	F	30	154
**8**	59	M	81	110
**Healthy**				
**9**	65	F		
**10**	59	M		
**11**	32	F		
**12**	56	F		
**13**	60	M		
**14**	54	M		

Supplementary Table 1: Demographics and clinical characteristics of patients with or without diabetic nephropathy.

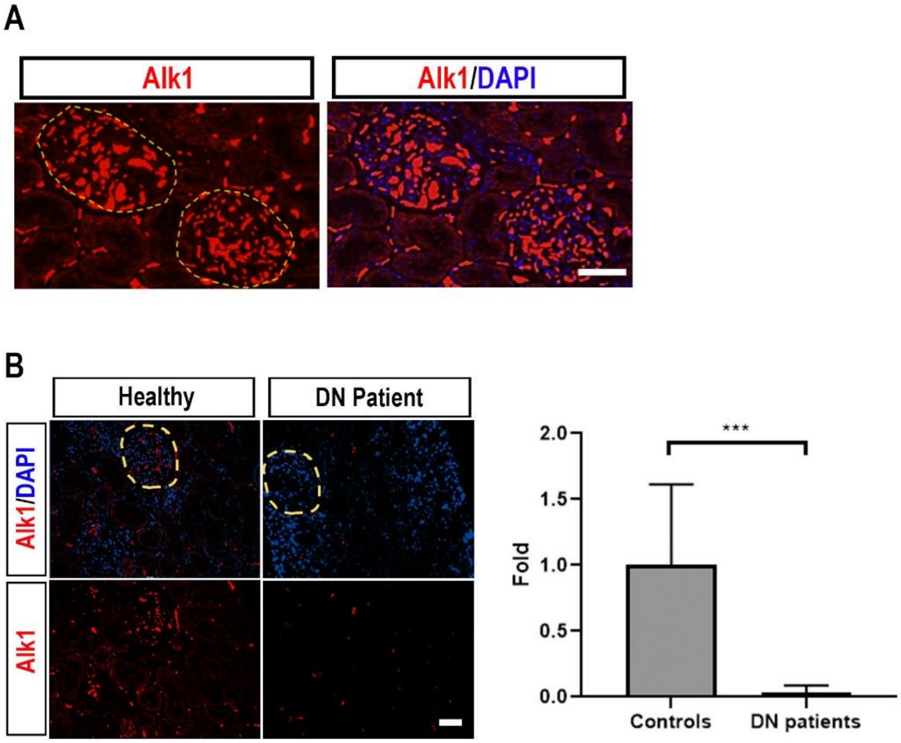

Supplementary Figure-1: Glomerular expression of Alk1 in control and diabetic nephropathy patients. A) Alk1 immunofluorescence in renal biopsy of control subject. B) Quantification of Alk1 glomerular immunofluorescence in renal biopsies of control subjects and diabetic nephropathy patients. Quantification was performed by assessing glomerular staining intensity for Alk1 of tissue samples from 6 healthy and 8 diabetic nephropathy patients. Scale bars: 100 µm.

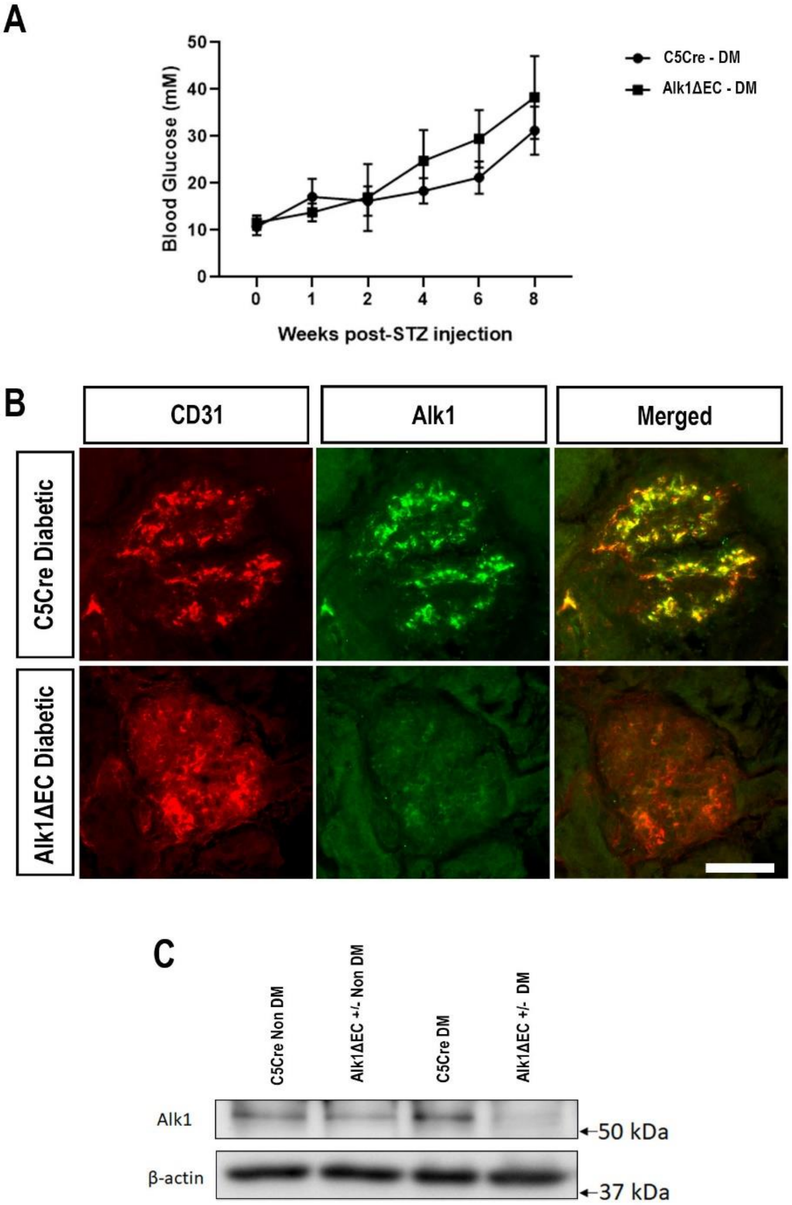

Supplementary Figure-2: Characterization of Alk1ΔEC+/- diabetic mice. A) Monitoring of blood glucose levels in C5Cre and Alk1ΔEC+/- mice following STZ injections. B) Expression of Alk1 in diabetic C5Cre or Alk1ΔEC+/- mice eight weeks following STZ injections. (C) Representative immunoblot of Alk1 expression in isolated glomeruli of control and diabetic C5Cre and Alk1ΔEC+/- mice. Scale bar: 25μm.

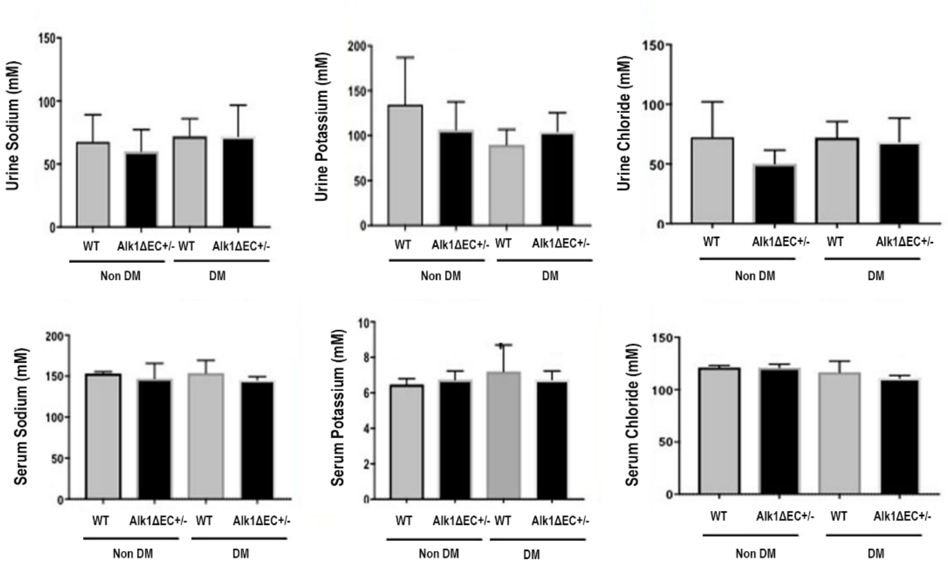

Supplementary Figure-3: Analysis of electrolytes in non-diabetic and diabetic C5Cre and Alk1ΔEC+/- mice eight weeks following STZ injections. (n=8 mice/group).

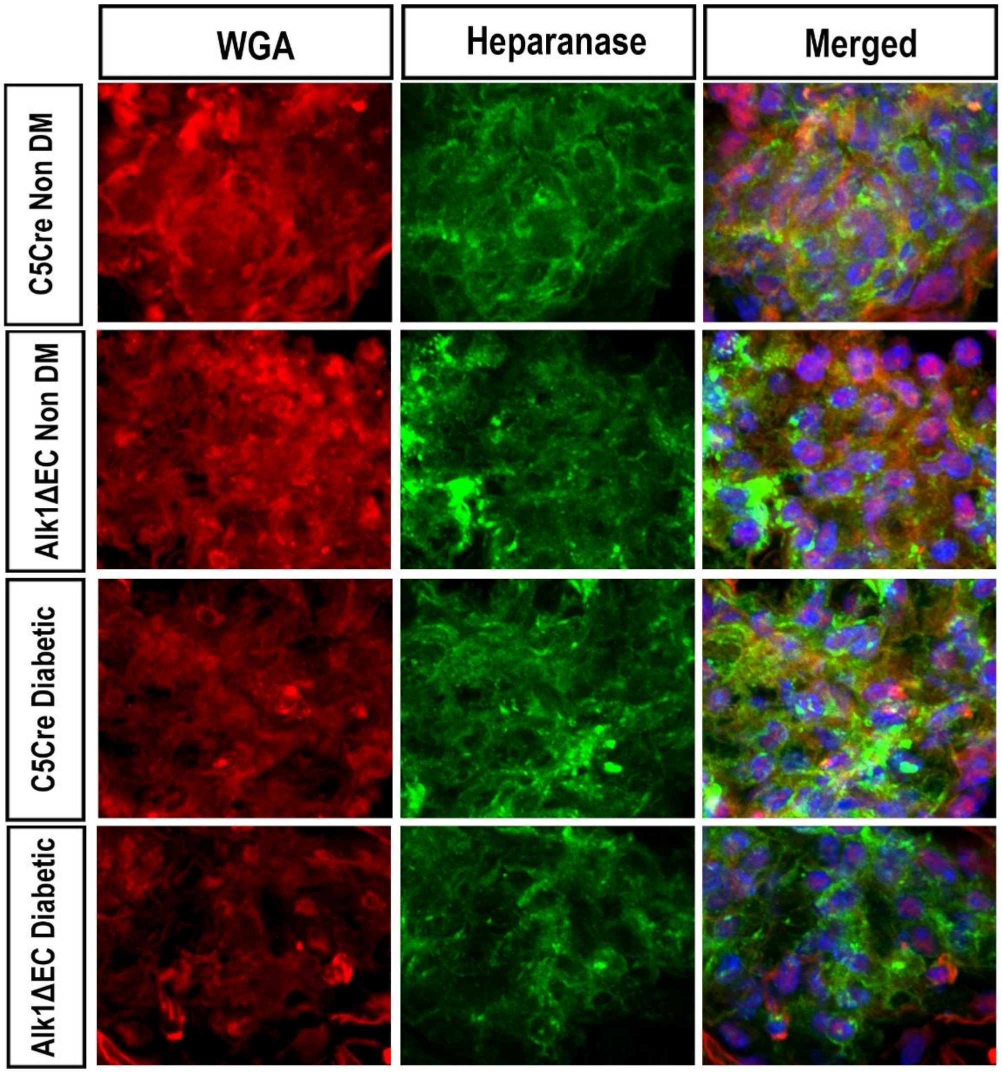

Supplementary Figure-4: Immunofluorescent staining with Wheat Germ Agglutinin (WGA; red), and anti-heparanase (green) on renal tissue from diabetic and non-diabetic C5Cre and Alk1ΔEC+/- mice eight weeks following STZ injections.

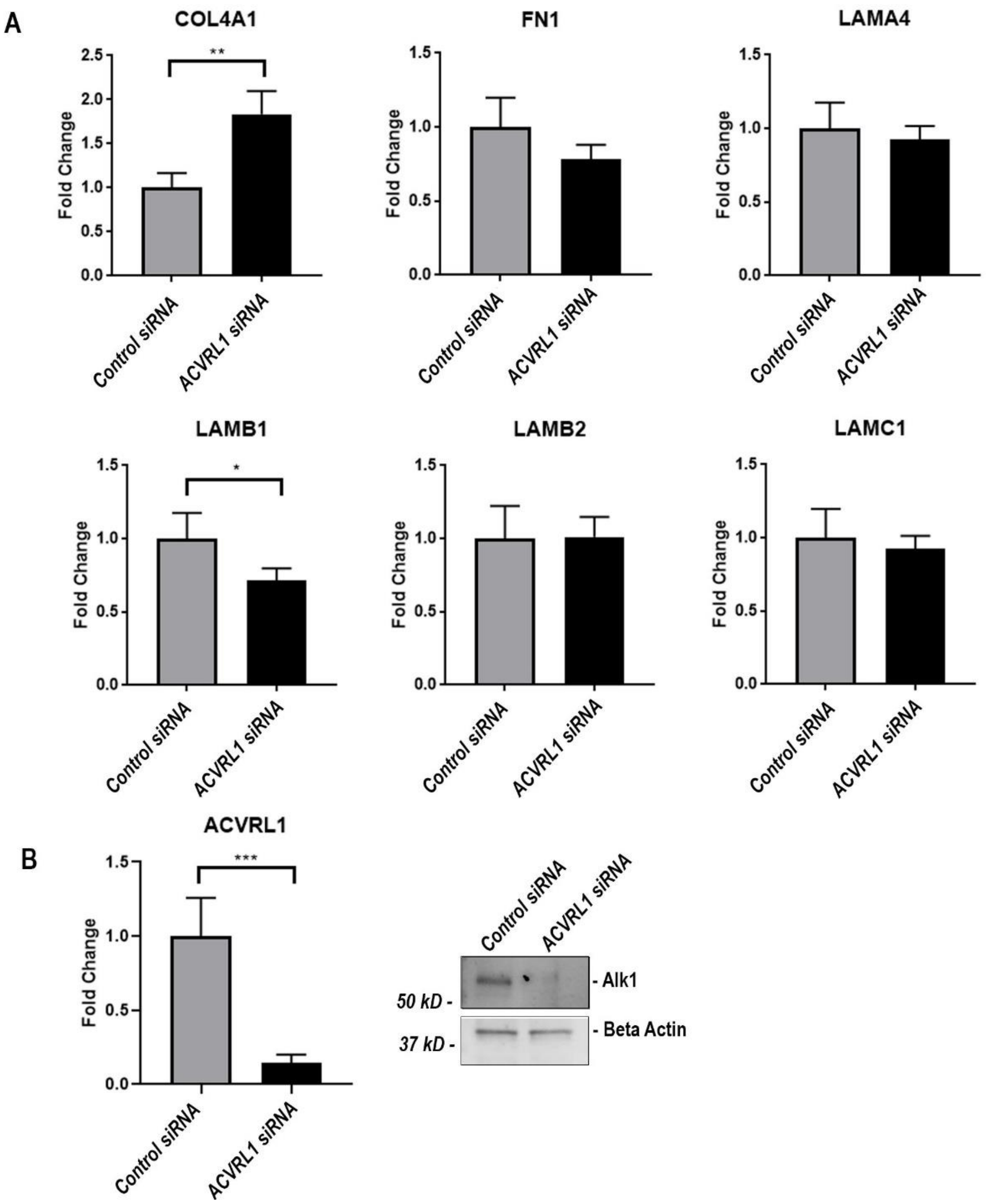

Supplementary Figure-5: Effect of Alk1 suppression on the expression of basement membrane genes. Human Umbilical Endothelial Cells (HUVEC) were transfected with scrambled or *ACVRL1* siRNA. (A) mRNA was harvested 48 hours after transfection and processed for quantitative PCR analysis for type IV collagen, fibronectin, laminin alpha4, laminin beta1, laminin beta2 and laminin gamma1. (B) Confirmation of decreased Alk1 expression in HUVECs transfected with *ACVRL1* siRNA by quantitative PCR and Alk1 immunoblotting. n=4 experiments done in triplicates. *p<0.05, ** p<0.01.

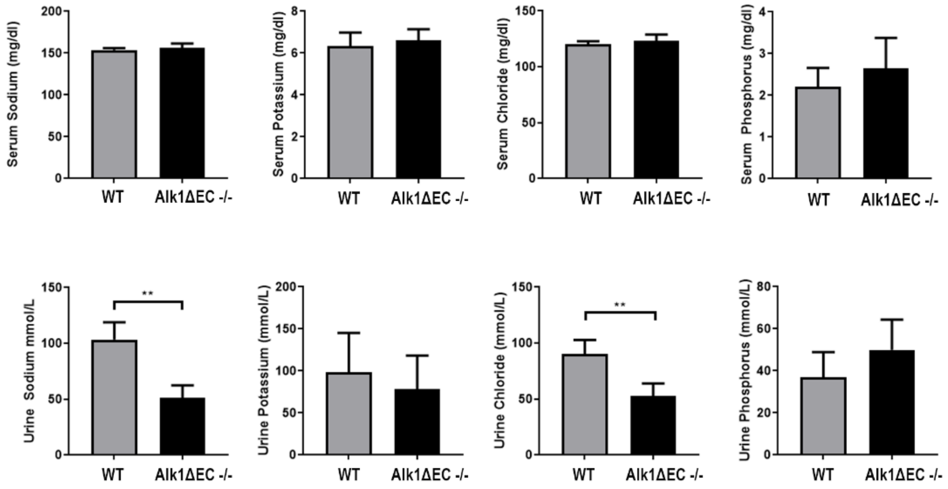

Supplementary Figure-6: Analysis of electrolytes in C5Cre and Alk1ΔEC-/- mice seven days after tamoxifen delivery. (n=6 mice/group). **p<0.01.
